# Intervertebral Disc and Adipokine Leptin—Loves Me, Loves Me Not

**DOI:** 10.3390/ijms22010375

**Published:** 2020-12-31

**Authors:** Goran Curic

**Affiliations:** Institute of Forensic Medicine, University of Bonn, Stiftsplatz 12, D-53111 Bonn, Germany; gcuric@uni-bonn.de; Tel.: +49-0-228-738344

**Keywords:** leptin, obesity, intervertebral disc, low back pain, inflammation

## Abstract

Leptin—the most famous adipose tissue-secreted hormone—in the human body is mostly observed in a negative connotation, as the hormone level increases with the accumulation of body fat. Nowadays, fatness is becoming another normal body shape. Fatness is burdened with numerous illnesses—including low back pain and degenerative disease of lumbar intervertebral disc (IVD). IVD degeneration and IVD inflammation are two indiscerptible phenomena. Irrespective of the underlying pathophysiological background (trauma, obesity, nutrient deficiency), the inflammation is crucial in triggering IVD degeneration. Leptin is usually depicted as a proinflammatory adipokine. Many studies aimed at explaining the role of leptin in IVD degeneration, though mostly in in vitro and on animal models, confirmed leptin’s “bad reputation”. However, several studies found that leptin might have protective role in IVD metabolism. This review examines the current literature on the metabolic role of different depots of adipose tissue, with focus on leptin, in pathogenesis of IVD degeneration.

## 1. Introduction

Our body changes as we age: the skin wrinkles, vessels and joints get stiffer, eye lens hardens, hair turns grey, and intervertebral discs degenerate. Some people age better than others, i.e., individuals differ in their aging trajectories [1]. An individual’s ageing pace is influenced by various genetic and environmental factors. The human body is exposed to environmental factors from the prenatal period onwards. While some environmental factors are almost invariable (e.g., climate factors), others are adjustable behaviors and life choices, often encompassed by the term lifestyle factors. The most common modifiable lifestyle factors include smoking, diet, alcohol consumption, and physical activity. Though ageing is a major risk factor in most diseases, lifestyle factors affect the course of probably all noncommunicable diseases such as ischemic heart disease, diabetes, cancer, and lumbar disc disease.

The number one cause of disability in the world is low back pain (LBP) [2]. As much as 84% of adults will suffer from LBP at some point during the lifetime (e.g., writing of this review was accompanied by an exacerbation of LBP), with approx. 10% being chronically disabled by LBP [3]. In the 25–49-year age group, LBP is ranked as the fourth most common cause of disability-adjusted life-years (after road injuries, HIV/AIDS, and ischemic heart disease) [4]. In the 10–24-year age group, LBP is ranked as the seventh most common cause of disability-adjusted life-years (after injuries, headache, depressive, and anxiety disorders). Hence, the disability and disease burden of LBP is enormous.

## 2. Low Back Pain and Lumbar Disc Disease

It is important to make a distinction between low back pain and lumbar disc disease. Low back pain is unpleasant subjective sensation and emotional experience, i.e., a symptom of an underlying condition. Typically, a pathoanatomical cause of LBP is unclear and the condition is termed a *non-specific* LBP. Lumbar intervertebral disc degenerative disease (LDD) is esteemed as the principal causative determinant of LBP [5]. Even in the presence of intervertebral disc (IVD) degeneration, an unambiguous nociceptive source remains mostly unclear [2]. A common definition describes that IVD degeneration is “an aberrant, cell-mediated response to progressive structural failure” [6]. The signs of IVD degeneration are not seldom findings in the young and healthy population [7]. The first morphological changes of lumbar discs are ascertainable among children and early adolescents—implying that “degeneration” is a somewhat inappropriate term [8]. Of note—even in the presence of LDD, the pain sensation is still classified as *non-specific* LBP. It was suggested that the term *degenerative disc disease* should apply only for degenerated disc causing pain [6], but the literature is inconsistent concerning the distinction of IVD degeneration and degenerative disc disease. From this perspective, in the following report, LDD is considered as degenerative changes of lumbar intervertebral discs evident radiologically or on advanced imaging (i.e., magnetic resonance imaging (MRI)), whereas alterations at microscopic or molecular level are termed IVD degeneration.

LBP and LDD are repeatedly associated with several shared risk factors, such as diabetes, smoking, obesity, and low levels of physical activity. However, the results and conclusions of the different studies regarding the association of LBP and LDD with different lifestyle factors are ambiguous. A recent seminal article from Freidin et al. on the genetic architecture of back pain and associated risk factors based on 509,000 individuals suggested the existence of at least two LBP-related molecular axes [9]. One molecular axis sheds light on the role of structural aspects of IVD and anthropometrics—with pleiotropic genetic effects (i.e., a phenomenon when one gene influences two or more seemingly unrelated phenotypic traits) underlying back pain, height, and IVD disturbances. The second axis points at the importance of pain perception and processing, with independent genetic correlations of back pain with depressive traits, neuroticism, and sleep disturbances [9].

Since LBP is an *illness* (i.e., *subjective* experience of symptoms), inter-individual differences in pain perception play an important role in LBP reporting [9]. Compared to LBP, LDD is a *disease*, i.e., *objectively* assessable condition. Therefore, when differences in imaging modalities and definitions of LDD are ignored, LDD represents a clinical entity somewhat easier to ascertain. IVD degeneration was observed at any vertebral level, with not necessarily matching risk factors, pathoanatomic findings, and treatment modalities [8]. In the lumbar spine, the degeneration is typically found at the two lowest IVDs: L4–L5 and L5–S1, representing vast majority of all degenerated discs.

Although LDD is a common disease, a common language to describe the problem is lacking. Different features of lumbar spine appear as the defining criteria of IVD degeneration—including disc height reduction, changes in disc signal intensity, disc bulging, disc herniation, disc irregularity, and anterior osteophytes [10,11]. Besides difficulties in defining LDD, other underlying methodological differences, including LDD grading systems (e.g., Pfirrmann or Thomas classification), imaging techniques (CT vs. T2 weighted MRI—suggested as the golden standard), and statistical methods, make the comparison of studies extremely difficult.

Excessive accumulation of adipose tissue is the most investigated lifestyle factor associated with the occurrence of LDD. This review aims to provide an overview of the role of adipose tissue in the development of degenerative changes of the lumbar intervertebral disc, with focus on leptin—the prototypical hormone produced by adipose tissue. The alterations of IVD architecture arise from changes in cellular metabolism of its cells. To understand the underlying pathophysiological disturbances at a molecular level, the physiology and pathophysiology of the intervertebral disc and adipose tissue are briefly reviewed.

## 3. On Intervertebral Disc

### 3.1. The Architecture of the Intervertebral Disc and Its Residents

**Figure 1:**
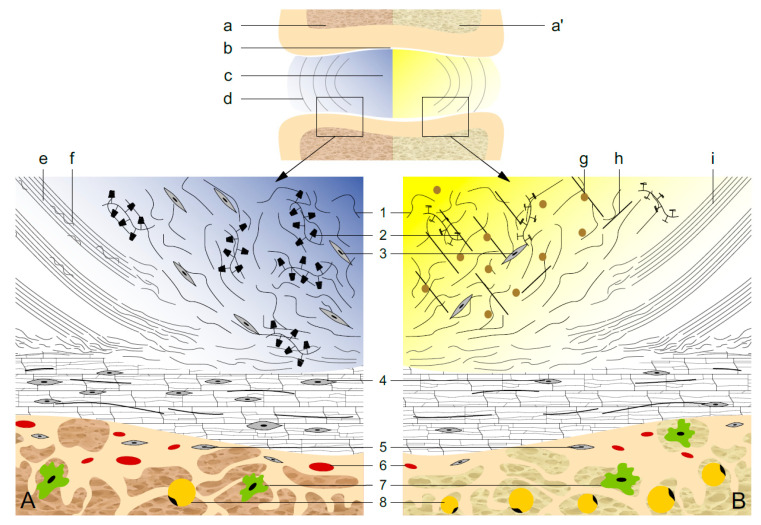
The intervertebral disc. Upper panel—the intervertebral disc flanked with adjacent vertebrae. Within the vertebrae is (**a**) red, hematopoietic bone marrow, or (**a’**) yellow bone marrow adipose tissue. The intervertebral disc consists of (**b**) bilayer of cartilage endplate and porous subchondral bony plate, (**c**) central semi-fluid nucleus pulposus, and (**d**) fibrous annulus fibrosus. Lower panel—scheme of (**A**) healthy intervertebral disc of a young individual with hematopoietic bone marrow, and (**B**) degenerated intervertebral disc with bone marrow adipose tissue. Nucleus pulposus is rich in (**1**) collagen type II and (**2**) water-attracting proteoglycans with attached glycosaminoglycans—depicted as bottlebrush structures. Depending on the age, nucleus pulposus contains between 70% and 90% of water (bluish color of the nucleus pulposus in panel A). The degenerated intervertebral disc (panel B), hallmarked with lower hydration, contains (**g**) accumulated advanced glycation end-products (yellowish discoloration). In the nucleus pulposus are its principal chondrocyte-like cells (**3**). Ageing disc (panel B) shows reduced cellularity, loss of (**1**) collagen type II, and increase in (**h**) collagen type I content. “Young” annulus fibrosus (panel A) in inner section contains collagen (**e**) type I and (**f**) type II, whereas degenerated annulus fibrosus (panel B) shows in (**i**) inner section loss of collagen type II. The cellularity of (**4**) cartilage endplate is decreasing with advancement of degeneration. In the subchondral bone are (**5**) osteoblasts, (**6**) capillary endings, (**7**) immune cells, and (**8**) adipocytes.

The intervertebral disc is a cartilaginous joint between two vertebrae. IVDs provide flexibility to the rigid spine and enable transmission of mechanical load. IVD consists of central gel-like nucleus pulposus (NP). Fibrous annulus fibrosus (AF) embraces nucleus pulposus laterally, while the endplates—a bilayer of the cartilaginous endplate (CEP) and a bony subchondral plate—frame the nucleus pulposus cranially and caudally (Figure 1). Besides structural role, CEP functions as a semi-permeable barrier that enables exchange of solutes and gases between the vertebrae and IVD matrix.

All three parts of IVD consist of water, proteoglycans, collagens (in NP mostly type II, and in AF mostly type I), non-collagenous proteins, and glycosaminoglycans (GAGs). The major proteoglycan is aggrecan—a large molecule aggregating with GAGs and stabilized by protein links. This complex creates large osmotic swelling pressure and attracts water—thereby creating a hydrated gel structure.

### 3.2. The Heterogeneity of IVD Cells

The cellular population of all three parts of IVD is heterogeneous. Within the extra-cellular matrix-rich NP of adults are its scant cells; small chondrocyte-like cells comprise less than 1% of its volume. However, until the age of four, the human IVD houses also large, vacuolated notochordal cells [12]. Gradual loss of cells with notochordal phenotype during early infancy coincide with complete loss of disk vascularization [8]. Recent works demonstrated that under light microscopy uniform NP cells differ in their transcriptional activity, e.g., regarding expression of collagen type II [13] or leptin receptor (LepR) [14].

However, the disappearance of notochordal cells in human IVD is a matter of debate, since a considerable proportion of cells within NP of adults display notochord-like phenotype [15]. Animal studies found that both NP cells and notochordal cells originate from the embryonic notochord [14]. Noteworthy, common model organisms for studying LDD—like mouse, rat, and rabbit—harbor notochordal cells within IVD into adulthood [8]. Since IVD represents rather isolated, immunologically privileged site [15], it is not exaggerated to imply that all NP cells originate from notochord. IVD might represent their niche for proliferation, differentiation, and senescence.

### 3.3. The Maintenance of IVD

The cells within IVD (as in any other the articular cartilage) are responsible for synthesis of macromolecules within the joint. IVD, as any other joint, undergoes aging process. The normal ageing process of IVD includes fragmentation and loss of proteoglycans (especially in NP) with a subsequent decrease in water content (Figure 1) [8]. At the same time, replacement of collagen type II with type I leads to further stiffening of IVD, while collagen cross-linking sugars is responsible for yellowish color of ageing disc (Figure 1) [8]. The degradation of cartilage endplate is evident, for example, as cartilage calcification and degradation of proteoglycan matrix. The aging IVD is characterized by decline in cell number and viability. However, clusters of cells are found within the IVD, implying local cell replication [16]. Because of its avascular nature, it is assumed that local production of regulatory mediators of cell proliferation is of great importance. Diverse anabolic and catabolic mediators diffuse through the IVD matrix [3].

IVD maintenance also includes controlled degradation of altered matrix macromolecules. The key “assignees” of IVD matrix degradation are the matrix metalloproteinases (MMPs) and related catabolic enzymes ADAMTS (a disintegrin and metalloproteinase with thrombospondin motifs), also called aggrecanases [17]. Major ADAMTSs in humans are ADAMTS4 or aggrecanase 1 and ADAMTS5 or aggrecanase 2. The proteoglycans (including aggrecan) are cleaved by both MMPs and ADAMTSs. Collagen type II is cleaved by MMPs: MMP−1, −2, −3, −8, −13, and −14 [8]. For example, human chondrocytes constitutively express and secrete MMP-13, but this catabolic enzyme is rapidly endocytosed, (together with aggrecanase-1 and the endogenous inhibitor of collagenases and aggrecanases type 3) and degraded [17]. In vitro study on bovine articular cartilage showed that cartilage degradation is reversible after cleavage of proteoglycans (by aggrecanase 1), but replenishment of collagen type II, after breakdown by MMPs (MMP-2 and -9), was demonstrated only after a short-term degradation [18].

Being the largest avascular tissue in the body, IVD nutrition relies primarily on the diffusion through the endplate (only the superficial cells of outer AF are supplied by extravertebral arteries) [19]. Therefore, it should be kept in mind that IVD cells are sparse and with diffusion-limited synthetic abilities. These biological constraints enable relatively slow matrix turnover. Within the given constraints, it is assumed that the matrix regenerative potential is limited. Hence, uncontrolled activation of MMPs in IVD matrix and/or absence of anabolic growth factors might represent the irreversible step in IVD degeneration.

## 4. Interpretation of Link between Obesity and Lumbar IVD Degeneration

The etiology of LDD is multifactorial—dependent on the interaction of several genes combined with one or more environmental factors. It is doubtless that age and specific gene variations (e.g., in MMP-genes) contribute to IVD degeneration, independent from obesity. The main hypotheses of association of obesity and lumbar IVD degeneration include (i) biomechanical overloading (“wear and tear”), (ii) disturbances in nutrition of IVD cells due to systemic alterations (e.g., atherosclerosis), and (iii) obesity-associated low-grade inflammation.

Excessive mechanical loading of the lumbar spine due to excessive body weight was a logical causative explanation of IVD degeneration. Numerous in vitro and in vivo models have shown that NP cells subjected to compressive loading alter expression of genes responsible for matrix maintenance (collagen type II, aggrecan, MMPs) and cytokines, e.g., interleukin (IL) 1β and tumor necrosis factor α (TNF-α) [20,21]. The stronger association of abdominal adiposity with LDD was interpreted with local over-loading due to increased abdominal mass. Though, the inference that the association of obesity with pain and degeneration is due to excessive loading of joints is contradicted with the occurrence of similar degenerative changes and associated pain of non-weight-bearing joints [22]. Therefore, mechanical loading alone is mechanistically insufficient to explain the link between fatness and LDD. Further research is necessary to understand the impact of mechanical overloading on the development of IVD degeneration [23].

Second, it is hypothesized that the link between fatness and the risk of IVD degeneration is due to the impact of obesity on development of general atherosclerotic and/or metabolic changes. Herewith it is assumed that an insufficient blood supply of the avascular IVD triggers degenerative changes. Both atherosclerosis and LDD advance with age and ischemic changes contribute to the development of LDD. However, the atherosclerotic changes of the arteries are found from the mid-20s, while IVD degeneration was already established in asymptomatic kids (mean age 12.5 ± 2.7 years) [24]. Therefore, when disregarding some rare genetic disturbances of lipid metabolism, it is unlikely that atherosclerotic changes or other metabolic changes of the arteries supplying the vertebrae could be so profound to cause IVD malnutrition in an otherwise healthy child.

Currently, the most alluring explanation of association between increased body fat and LDD is due to the endocrine role of the adipose tissue. Excessive accumulation of adipose tissue, considered in its entirety, is a disease characterized by dysfunction of multiple tissues and organs, including adipose tissue, liver, brain, and pancreas. Underling disturbance is subclinical chronic inflammation.

## 5. On Fatness

Since normal means “being of the sort or kind that is expected as usual, ordinary, or average” and implies “lack of deviation from what has been discovered or established as the most usual or expected” [25], being overweight is becoming another normal body shape. Namely, overweight is defined as body mass index (BMI; weight in kg per height squared) greater than or equal to 25 kg/m^2^, and obesity as BMI greater than or equal to 30 kg/m^2^; in 2016, 39% of adults were overweight, and 13% obese [26].

Excessive accumulation of adipose tissue results from an imbalance between energy intake and expenditure. Excessive body fat is associated with dyslipidemias (e.g., high LDL (low-density lipoprotein) cholesterol, low HDL (high-density lipoprotein) cholesterol, high level of triglycerides), hypertension, and insulin resistance—a common forerunner of diabetes. Although dyslipidemia, hypertension, and insulin resistance occur also as isolated conditions, the body of literature studying the association of LDD these with health conditions in absence of increased body weight is scarce.

The meta-analyses are unanimous that overweight and obesity are associated with increased risk of both low back pain [27] and lumbar disk disease [28]. However, most studies on LBP and LDD do not allow drawing conclusions on the role of fatness, instead, on the role of body weight adjusted for height, i.e., BMI—as a proxy of body fatness.

### 5.1. Defining the Problem—Adiposity Indices

There are several methods aiming to assess the quantity of body fat: from a simple BMI to methods requiring specialized equipment, e.g., MRI or dual energy X-ray absorptiometry. Expensive and time-consuming methods are mostly reserved for research settings with not so large number of participants. Therefore, BMI is prevailing, often exclusively assessed measure of obesity in studies about LBP and LDD.

BMI is far away from an ideal index of obesity. While BMI does adjust for height, it does not adjust for what makes up the weight—e.g., it cannot distinguish the weight of muscle from the weight of fat. Since the humanity is faced with epidemic of obesity and not epidemic of muscularity, this shortcoming is of minor importance. Far more worrisome is the fact that BMI does not account for the distribution of body weight, i.e., insufficiency of BMI as a biomarker of abdominal adiposity. Moreover, BMI does not account for age, gender, and ethnic differences. Therefore, World Health Organization recommends different BMI cut-off values for overweight and obesity in the Asian populations [5,29].

Recent international Consensus Statement on cardiometabolic risk associated with increased adiposity concluded that BMI alone is not sufficient to assess obesity-related health risks [29]. The main recommendation is to measure the waist circumference routinely, as a surrogate index of abdominal obesity—since it “provides both independent and additive information to BMI for morbidity and mortality prediction” [29]. When defining abdominal obesity in different ethnic groups, the International Diabetes Federation recommends using different cut-off values of waist circumference. However, waist circumference is also not an ideal index of abdominal obesity. As a simple waist perimeter, it is unable to provide a distinction between “good” subcutaneous adipose (SAT) and “bad” visceral adipose tissue (VAT), the latter being associated with more adverse health risk.

Another methodological issue regarding the indices of adiposity needs to be addressed—the way in which the data is being analyzed. In the vast majority of LBP and LDD studies, the BMI and waist circumference, as continuous variables, are converted to broad categorical variables. Herewith a lot of important information is lost and such data impoverishment partly explains the inconsistencies between the studies. For example, the full magnitude of effect of increased waist circumference on all-cause mortality and morbidity is first evident after adjustment for BMI—when both variables are considered as continuous [29]. In plain language, when assessing the association of a clinical entity (e.g., LDD or LBP) with waist circumference and BMI as continuous variables, “for a given waist circumference, the higher the BMI the lower the adverse health risk” [29].

### 5.2. Left Undefined

Up to 50% of population falls into these two categories with obesity indices poorly defined: metabolically healthy obese and metabolically unhealthy normal weight [30,31,32].

#### 5.2.1. Metabolically Unhealthy Normal Weight

Up to 30% of normal weight adults have an accumulation of adipose tissue in the truncal area, with waist circumference not necessarily larger than adults with similar BMI [32]. Thus, these individuals exhibit one or more metabolic abnormalities that typically accompany obesity (e.g., hypertriglyceridemia, impaired fasting glucose, hyperinsulinemia) and have an increased risk of diabetes and heart disease [32]. There is a lack of consensus in referring this phenotype—with metabolically unhealthy normal weight (MUNW) being one of the terms. The hallmark of MUNW phenotype is fat accumulation as abdominal subcutaneous and visceral depots, and in the liver, but not in skeletal muscle. Often is MUNW phenotype also characterized with lower cardiorespiratory fitness and muscle mass [32]. A recent study on 1114 individuals suggested that higher intakes of potassium, calcium, magnesium, and vitamin A (i.e., nutraceuticals) reduce the risk of development of unhealthy phenotype in normal weight adults [33].

#### 5.2.2. Metabolically Healthy Obese

At the other end of the spectrum are individuals who account for about a third of BMI-defined obese—having excessive body adipose tissue but favorable metabolic profile. The phenotype is referred to as “metabolically healthy but obese” (MHO) and the phenomenon as “the obesity paradox” [31]. Metabolic “health” is defined as absence of hypertension, elevated blood triglyceride and increased fasting glucose levels, and normal HDL levels [30,31]. Underlying genetic and environmental factors are poorly understood, but literature implies at differences in VAT accumulation [34,35] and adipose cell differentiation [36].

A study on postmenopausal obese women showed that MHO participants had on average 49% less abdominal visceral fat (measured by CT) [34]. In a study on adults of both sexes and compared to “metabolically abnormal obese,” Stefan et al. found that MHO individuals of both sexes had markedly less liver fat (60% in women, and 46% in men) and MHO men had 43% less ectopic fat in soleus muscle [35]. A study on equally obese, otherwise healthy participants differing in insulin sensitivity, showed that insulin resistance was associated with a higher ratio of small to large cells in the subcutaneous periumbilical adipose tissue [36]. Moreover, insulin sensitive individuals, representing the MHO phenotype, showed two- to three-fold higher expression of adipose cell differentiation genes (*inter allia* glucose transporter type 4 and adiponectin). From other adipokines, the authors only assessed expression of adipsin/complement factor D [36]. Recent studies on murine model of MHO implied that adipokines leptin and resistin might be important determinants of MHO phenotype [31,37].

### 5.3. Adipose Tissue Depots

According to its localization in the body, we recognize (i) subcutaneous and (ii) visceral adipose tissue, and (iii) ectopic fat depots, while (iv) bone marrow adipose tissue is considered as a depot *sui generis.*

The largest adipose tissue depot is subcutaneous adipose tissue, representing more than 80% of total body fat—with abdominal, gluteal, and femoral depot being characterized the best. Most visceral adipose tissue is located within the abdominal cavity, including omental, mesenteric, and retroperitoneal (perirenal) depots. Intra- and retro-peritoneal depots represent 10–20% of total body fat in men and 5–10% of total fat in women—underlying the difference between typical “male” (android, central, or truncal) and “female” (gynoid, gluteofemoral or peripheral) fat distribution [38]. Adipose tissue is present in numerous discrete anatomical depots that affect the function of adjacent tissues and organs, e.g., perivascular, epi- and pericardial, gonadal, and infrapatellar fat.

Ectopic fat is termed as deposition of excess fat in tissues not classically presenting macroscopic clusters of fat tissue or normally not present at some locations. Ectopic fat includes depots in liver (“fatty liver”), heart, pancreas, renal sinus, and skeletal muscles (as interspersed intramuscular fat). A small study based on lumbosacral liposuction gave an insight on the adverse impact of ectopic intramuscular fat [39]. Liposuction of ectopic fat from atrophied paravertebral muscles was associated with clinical improvement of LBP in 77.5% patients (31/40; mean age of 58 years). The approach did not gain much attention in the literature nor practice.

An interesting small fat depot is extradural adipose tissue within the spinal canal—termed epidural fat. This unencapsulated fat lies between posterior longitudinal ligament and spinal dura and its volume increases with increasing volume of adipose tissue. It was demonstrated that epidural expresses TNF-α, IL-1β, IL-6 IL-8, and adiponectin [40,41].

An interesting, though small, study on healthy monozygotic male twins in mid-30s [42] pointed at the differential regulation of fat depots. The twins were discordant for leisure time physical activity within the past three years. The inactive twins had on average 41% more intraperitoneal fat mass, while differences in volume of subcutaneous abdominal fat and BMI were insignificant (13% and 3%, respectively) [42].

### 5.4. Types of Adipose Tissue and Its Cells

Most of all adipose tissues in humans are white adipose tissues (WAT), independent of the depot, in both VAT and SAT. The principal cells are white adipocytes, which make ~90% of the tissue volume. The hallmark of white adipocytes is a single lipid droplet occupying about 95% of the cell volume. The size, metabolism, and function (e.g., in lipolysis, triglyceride synthesis and storage, and adipokine portfolio) of white adipocytes differ in relation to the depot.

Adipose tissue houses many other cell types, including endothelial cells, preadipocytes, pericytes, immune cells (e.g., macrophages, lymphocytes), and multipotent stem cells. “Others” outnumber the adipocytes: 1–2 million adipocytes vs. 4–6 million other cells per gram of human adipose tissue [38]. Furthermore, different depots show distinct cell types with different adipokines’ expression profiles [38].

Adipose tissue characterized with adipocytes containing numerous smaller lipid droplets and brownish mitochondria is brown adipose tissue (BAT) [43]. BAT in adults is found in small depots (in cervical-supraclavicular, paravertebral, and perirenal region) and accounts for approximately 10% of the body fat. BAT has a crucial role in cold-induced thermogenesis, but it is also an active endocrine organ—it releases mediators affecting the function of white adipose tissue and many other organs [43].

Within bone marrow are adipocytes, distinct from those in white and brown adipose tissue, and sometimes referred to as beige adipocytes. The quantity of bone marrow adipose tissue (MAT) increases continuously during the lifetime and by the age of 25, 70% of hematopoietic (red) bone marrow is replaced by “yellow” MAT [19]. MAT produces numerous signaling molecules including adipokines (e.g., leptin and adiponectin), insulin-like growth factor 1 (IGF-1), and pro-inflammatory cytokines [44]. MAT depots are also heterogeneous in their nature. A study in a broad adult population showed that lower quantity of vertebral MAT, but not femoral MAT, was associated with regular physical activity, independent of age, gender, or waist circumference [45]. Increasing volume of VAT, independent of the volume of SAT and level of physical activity, was associated with increased vertebral MAT [46]. The same cross-sectional study showed independent associations of increased vertebral MAT with increasing age, hemoglobin A1c, blood triglycerides, and LDL [46].

### 5.5. Adipose Tissue Derived Mediators

The primary role of the “major” cells in adipose tissue—the adipocytes—is energy storage and utilization under the orchestration of hormones like insulin and glucagon and by sensing the energy needs of the body via lipids in blood. The adipocytes and other cells in the adipose tissue also secrete hormones and “communicate” with other organs, including brain, gastrointestinal tract, pancreas, and muscle [43,47,48,49]. Hence, adipose tissue is the largest endocrine organ in humans.

White, beige, and brown adipocytes physiologically synthesize and release numerous peptide hormones (adipokines), bioactive lipids (lipokines), and exosomal microRNA molecules [49]. These mediators regulate numerous physiological processes, such as appetite control, glucose and lipid homeostasis, and inflammation. The most investigated adipokines are leptin, adiponectin, resistin, and chemerin. The term adipokine is misleading since many adipose tissue-derived mediators are also produced in other organs and tissues. Adipose tissue also secretes peptides known as “general” mediators, e.g., inflammatory cytokines, such as IL-1, IL-6, and TNF-α [3,19,47,49]. Therefore, excess of body fat often equals more inflammation.

Albeit adipocytes physiologically synthesize and release numerous mediators, many initially described adipokines are not secreted by adipocytes, but by “others”—other cells within the adipose tissue (e.g., endothelial and immune cells) [49]. Although adipose tissue harbors numerous cell types and must be observed as a whole and in relation to the adjacent tissues, it seems that its resident macrophages are particularly important. The importance of resident macrophages in regulating tissue homeostasis in obesity is reviewed elsewhere [50,51].

Visceral adipose tissue is especially active in the production of mediators. VAT is strongly associated with obesity-related metabolic disturbances and several risk factors (e.g., cardiovascular risk, cancer)—hereby influencing other tissues (and IVD) in endocrine fashion [52].

Bone marrow adipose tissue is an example of endocrine and paracrine acting adipose tissue depot. MAT releases leptin and other mediators (adiponectin, cytokines, and lipids) that exert different local (hematopoiesis, bone remodeling) and systemic effects [53]. Because of its proximity to IVD, vertebral MAT imposes itself as the major source of adipokines that may affect IVD metabolism in paracrine fashion [19]. However, the adipokines from other adjacent fat depots like epidural and paraspinal fat, may affect IVD homeostasis.

## 6. On Leptin

Leptin is the “first-born” of the adipose tissue—this hormone was discovered in 1994 and it changed our understanding of adipose tissue as an inert fat storage tissue [47]. Leptin is mostly produced in white adipose tissue, but also by other types of adipose tissue (MAT and BAT) and many other tissues, including enterocytes, cartilage, and IVD cells [44,54,55,56,57]. Leptin blood level is approximately proportional to the body fat stores—somewhat like a reserve fuel sensor in the car—herewith playing a crucial role in the control of energy homeostasis and body weight.

Leptin has central “anorexigenic” effects: it inhibits energy intake by hypothalamic regulation of appetite and increases energy expenditure by activating its receptors (LepR) on target cells [58,59]. Mutations in the leptin or LepR gene result in severe, early onset obesity, both in animal models and in humans [60]. Moreover, obesity is characterized by diminished leptin receptor signaling, designated as *leptin resistance* [59]. In leptin resistance it is mostly unclear, how leptin intracellular signaling is altered (e.g., disturbances of LepR and corresponding cytosolic pathways).

Leptin has important role in innate immunity. It mediates signaling of, *inter alia*, macrophages, monocytes, and dendritic cells, and stimulates production of mostly pro-inflammatory cytokines. The cells of adaptive immunity (e.g., immune cells within thymus and spleen, regulatory T cells) are also leptin-responsive [61]. Hence, excessive fat tissue leads to hyperleptinemia and subsequent systemic inflammation. Leptin is assumed to be the main contributor to inflammatory and pain-related effects in obesity [55].

### 6.1. Nutraceuticals, Fat Tissue, and Leptin

Since health burden of obesity and related disorders (e.g., diabetes, metabolic syndrome) is enormous, it is not surprising that there are many therapeutic approaches aiming at treating obesity-related metabolic disturbances. Aside from surgical, behavioral, and pharmacological approach [62], an interesting arm of the research is focused on the potential of natural compounds (nutraceuticals). A recent study implied that “good food” might protect the metabolically healthy obese individuals, while “bad food” might boost the development of “metabolic unhealthy traits” in metabolically unhealthy normal weight individuals [33].

A synthetic structural analog of glabridin (HSG4112)—a polyphenolic compound extracted from licorice roots (*Glycyrrhiza glabra* L. (*Fabaceae*)—is promising anti-obesity drug, currently in clinical trial for obesity treatment [63]. A six-week administration of the analog caused return to normal body weight by high fat diet-induced obese male mice. The treatment also led to the reduction of VAT and normalization of serum leptin, insulin, and glucose levels [63].

Lunasin—a plant peptide found in soybeans and some cereals—is among the most studied dietary bioactive peptides [64]. A study on high-fat-diet-induced obese mice pointed once more on differential regulation of adipose tissue between the sexes—lunasin had lowering effect on leptin serum levels only in females [64]. Another natural compound—salidroside from *Rhodiola rosea*—was tested in a similar experimental setting (mice fed on high-fat diet) [62]. A 48-day treatment with salidroside induced fat mass reduction (liver, epididymal WAT) and “greatly attenuates inflammation in WAT.” Moreover, salidroside-treated mice showed sharp decline in plasma leptin levels and improved glucose homeostasis, the latter is primarily due to activation of leptin signaling transduction in hypothalamus [62].

Since obesity and diabetes are mostly two sides of the same coin, it is not surprising that nutraceuticals and “pharmaceuticals” cause alterations in shared metabolic and genetic pathways. Many other nutritional interventions (caloric restriction, prebiotic, probiotic, and polyphenol supplements) showed leptin decreasing effect ([65], and referenced therein) or antagonize leptin adverse effects in vitro [66]. However, the molecular background of nutritional interventions on leptin signaling is almost completely unknown. In addition, no study observed the LDD or IVD degeneration in the light of dietary intervention via natural compounds. The commonness of LDD and a flood of nutraceuticals on the global market, as well is potential underlying role of nutraceuticals in metabolic health call for research efforts in this field.

### 6.2. Leptin Signaling

The circulating leptin may affect IVD homeostasis in an endocrine fashion directly, by signaling through its receptors, and indirectly—through the action of leptin-induced cytokines. The infrapatellar fat pad is an illustrative example of multipotency and depot-specificity of adipose tissue. It serves as a shock-absorbing cushion of the knee joint and a major source of adipokines in the synovial fluid (the rest is synthesized by other cells within the joint) [67]. Level of leptin in synovial fluid of osteoarthritis patients undergoing total knee replacement surgery positively correlated with the levels of MMP-1 and MMP-3 [68]. Knee joint cells in patients with and without osteoarthritis showed expression of LepR [69]. Leptin alone, and in combination with pro-inflammatory IL-1β, stimulated production of catabolic enzymes—MMP-1, MMP-3, and MMP-13 in vitro [68].

#### Leptin Receptors and Intracellular Pathways

**Figure 2:**
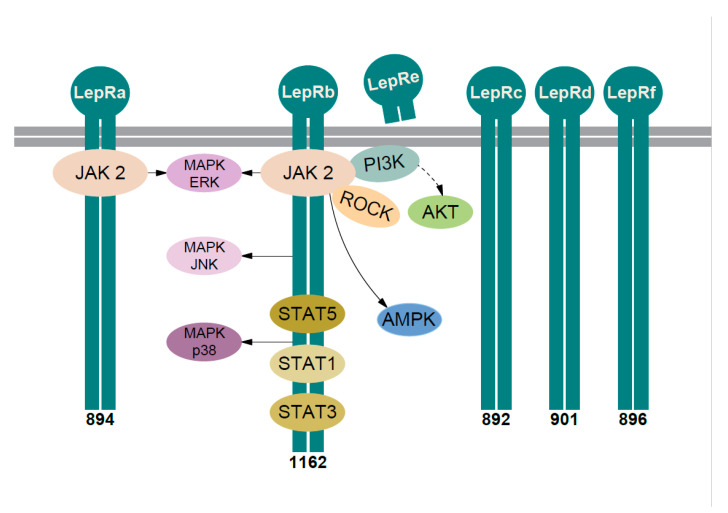
Leptin signaling pathways. Six isoforms of the leptin receptor are obtained by alternative splicing and named LepRa–LepRf. All isoforms share identical extracellular binding and transmembrane domain. LepRe is the soluble receptor isoform—a cleavage product of the extracellular domain of the LepRb. Five isoforms—LepRa–LepRd and LepRf—are bound to the cell membrane (depicted as two thick gray lines). Their intracellular domains differ—the number below isoform indicates the number of amino acids in the intracellular domain. LepRb possesses the longest intracellular domain and contains motifs for the complete activation of the JAK2/STAT pathway. After leptin binds the LepRs they oligomerize (here depicted only as dimerization) and activate associated JAK2. The autophosphorylated JAK2 can lead to the activation of PI3K/AKT, ROCK (Rho/ROCK), and AMPK pathway. JAK2 phosphorylates LepRb, the three important tyrosine residues in the cytoplasmic tail (Tyr985/1077/1138). Via phospho-Tyr985 activated at least three MAPK pathways: ERK, JNK, and p38. STAT5 binds to phospho-Tyr1077 and phospho-Tyr1138, while STAT1 and STAT3 bind to phospho-Tyr1138. LepRa also activates JAK2 and MAPK-ERK pathway. Solid arrow—one or more proteins in the pathway. Dashed arrow—more proteins in the pathway. LepR—leptin receptor, JAK2—Janus-activated kinase 2, STAT—signal transducer and activator of transcription, PI3K—phosphatidylinositol 3-kinase, AKT—Protein kinase B, also known as Akt, ROCK—Ras homolog gene family/Rho-associated coiled-coil-forming protein kinase, AMPK—adenosine monophosphate-activated protein kinase, MAPK—mitogen-activated protein kinase, ERK—extracellular signal-regulated kinase, JNK—c-Jun-N-terminal kinase. See text for more details.

Leptin has one soluble plasma receptor (LepRe) and at least five membrane-bound receptors (LepRa, LepRb, LepRc LepRd, and LepRf), the latter differing only in cytoplasmic domain (Figure 2) [60,69]. The roles of the short isoforms (with short cytoplasmic domain: LepRa, -c, -d, and -f) remain to be elucidated [70]. Of note, the expression of short isoforms is not “marginal,” e.g., LepRc is as abundant or more prevalent than LepRa in some tissues ([71] and referenced therein). The soluble isoform is released after the cleavage of the extracellular domain (Figure 2) and it is one of the proteins that modulates the activity of plasma leptin [60,72].

LepRa is the most common isoform, expressed in most tissues, often co-expressed with LepRb. In vitro studies showed that leptin via LepRa induced Janus-activated kinase 2 (JAK2) pathway with the activation of the extracellular signal-regulated kinase (ERK) (Figure 2), albeit weaker—compared to LepRb [60]. There are contradictory data on the role of LepRa in transport of leptin through the blood-brain barrier; hence, its role is still mostly unknown [60,71].

LepRb is the long isoform associated with “full” signaling capacity, mainly found in the central nervous system, with most intensively studied role in hypothalamic neurons. The receptor mediates signal through at least five distinct signaling pathways (Figure 2): (i) JAK2/signal transducer and activator of transcription (STAT) pathway, (ii) mitogen activated protein kinase (MAPK) pathways, (iii) adenosine monophosphate-activated protein kinase (AMPK) pathway, (iv) phosphoinositide 3-kinase (PI3K) pathway, and (v) Rho/ROCK (Ras homolog gene family/Rho-associated coiled-coil-forming protein kinase) pathway [60,71,73].

The best described is signaling via JAK2/signal transducer and activator of transcription (STAT) pathway [60,71]. The activation of canonical JAK2/STAT3 pathway leads to the modulation of several STAT3-responsive genes. It was demonstrated that leptin-activated JAK2/STAT3 pathway is crucial for acute hypothalamic appetite reduction and acute liver glucose metabolism [74]. A study on neuron-specific STAT3 knockout mice showed that this pathway was crucial (but not the only pathway) for leptin signaling in hypothalamic neurons ([60], and referenced therein). LepRb-JAK2 signaling activates also other STATs in vitro—STAT1, STAT6, and STAT5 (also in vivo) [60], pointing at the complex fine-tuning of leptin intracellular signaling via LepR/JAK2 complex.

Hypothalamic LepRb-JAK2 pathway also induced MAPKs, AMPK, and PI3K pathway—involved in the control of appetite, glucose homeostasis, and development of obesity [60]. Signaling through these multiple intracellular pathways was shown mostly in central nervous system, but it was demonstrated also in IVD and other tissues (e.g., liver cells and muscle tissue) [60,72].

In IVD cells it was shown that leptin signaling (Figure 2) is mediated also through AMPK, PI3K, Rho/ROCK pathway as well as via three distinct subgroups within the MAPK family: ERK1/2, p-JNK (c-Jun-N-terminal kinase), and p38 [60,71,73,75].

The study on cultured HeLa cells expressing LepRa and LepRb has resulted in some interesting findings [71]. Majority of LepR (80–90%) were found in the intracellular compartments, and both isoforms have short half-life at the cellular surface [60,71].

### 6.3. Leptin and Leptin Receptors in IVD in Intervertebral Disc

#### 6.3.1. Human Studies—Direct Evidence In Situ

Considering the quantity of data investigating the role of leptin and leptin receptors on IVD homeostasis wonders that leptin was directly demonstrated within human IVD (in situ) in only three studies (Table 1). Leptin protein was proven in situ once in AF—by Gruber et al. [54], and twice in NP—in studies by Zhao et al. [55] and Hsu et al. [76]. One study analyzed leptin protein in human AF tissue extracts [77]. Three studies showed *LepR* in human IVD in situ [14,54,55].

Gruber et al. analyzed AF from 28 patients (disc herniation and degenerative disc disease) and seven young control subjects (aged newborn to 10 years) [54]. In control group was immunostaining for LepR positive for all AF cells, but negative for notochordal cells in NP. The staining of AF from patients with LDD was positive just for some cells (both single and clustered cells). The IVD of young donors showed uniform staining for leptin, while degenerated IVDs showed the staining with similar pattern as for LepR (regarding single and clustered cells). Of note, Gruber et al. could not distinguish isoforms of leptin receptor.

Zhao et al. studied the surgical samples from 45 patients with lumbar disc herniation (aged 18–74 years, mean 48 years) [55]. They showed that the percentage of leptin- and LepR-positive cells in NP slightly increased with advancing age (with range ≈ 5–30%, and ≈5–25%, respectively). The positive cells were frequently clustered and located in areas showing proliferation. Zhao et al. detected “functional leptin receptor,” i.e., it is unclear which isoform(s) were assessed [55].

On IVDs from 91 patient with LDD (mean age 58 years), Hsu et al. showed that BMI positively correlated with histologic degeneration score, plasma leptin level, and ratio of leptin and MMP-1 immunostaining grade (adjusted for age and sex, univariate analysis) [76]. However, multiple linear regression analysis showed that only MMP-1 immunostaining grade was independently associated with IVD histologic degeneration score (in both cervical and lumbar segments) [76].

Koerner et al. assessed the anterior and posterior section of AF from patients undergoing surgery because of LDD (mean age 43.3 years) [77]. In AF extracts, 10 (of the 42 assessed) growth factors and cytokines were increased in the posterior section of AF (*inter allia*—leptin, IL-4, IL-5, IL-6, TNF- β, and IGF 1).

Gao et al. studied NP cells from 20 patients with degenerative disc disease or scoliosis (mean age 36 years) harvested during microendoscopic discectomy [14]. They demonstrated that the percentage of LepR positive cells decreased with increasing grade of IVD degeneration—from 75% in Grade II (Pfirrmann MRI-based five-graded classification system of lumbar IVD degeneration) to 32% in Grade Ⅴ [14]. Of note—Zhao et al. established increased percentage of LepR positive cell with increasing age [55].

#### 6.3.2. Cultured Human IVD Cells

Five studies assessed the leptin signaling in cultured human IVD cells (Table 1).

A study on NP from 45 patients IVD degeneration and radiculopathy (mean age 48 years) showed that both LepRa and LepRb messenger ribonucleic acid (mRNA) were widely expressed in NP, whereas expression of LepRb mRNA correlated with BMI [78]. A culture of NP cells from eight patients (Thompson degeneration Grade 2–3 in Thompson five-graded classification system of lumbar IVD degeneration based on gross morphology) showed that leptin induced cytoskeleton reorganization with change of cell shape [78]. Another study also demonstrated that leptin stimulation of cultured NP cells causes cytoskeletal reorganization and appearance of “epithelioid morphology with increased cellular spreading” [75]. The NP cells originated from seven patients with LLD (mean age 35 years, Pfirrmann Grade III) and the effect included activation of Rho/ROCK intracellular pathway [75].

Culture of NP cells from eight LDD patients (aged 31–40 years) showed that NP cells expressed both LepRa and LepRb [79]. Stimulation with leptin induced activation of three intracellular signaling pathways (STAT3, AKT, and ERK1/2)—as early as 5 min after leptin stimulation—with consequential cell proliferation (i.e., upregulation of cyclin D1—an important regulator of cell mitosis). After 24 h serum deprivation (to model of nutrient deficiency), leptin-treated NP cells showed increased viability. The authors suggested that leptin enables survival of NP cells under circumstances of nutrient shortage [79].

Sun et al. studied the *ligamenta flava—*paired elastic ligaments running between adjacent laminae of the vertebral bodies (at level L4/5)—hence, not intervertebral disc directly [80]. They found higher leptin mRNA and protein expression in ligamenta flava of 12 patients with lumbar spinal canal stenosis compared to 12 patients with lumbar disc herniation (mean age 58 years and 63 years, respectively). After leptin stimulation of cultured cells of ligamentum flavum, they demonstrated elevated expression of collagen type I and type III (as fibrosis markers), and IL-6, the latter via nuclear factor-κB pathway [80].

In a study on cultured NP from four scoliosis patients (*not* LDD) leptin decreased the expression of aggrecan and induced the expression of aggrecanases via p38-MAPK pathway [81]. Hence, the study implied that leptin has catabolic effect on IVD.

### 6.4. Leptin Signaling in Human Bone and Cartilage

The regulation of bone metabolism by leptin is both central, through its activity in central nervous system, and local, through LepR on many cells within bone niche. Numerous studies have showed direct anabolic effects of leptin on bone and cartilage via LepR on both osteoblasts and chondrocytes in vitro and in vivo ([82], and referenced therein).

In a culture of chondrocytes obtained by knee biopsy after injury, Figenschau et al. showed that leptin acts directly on chondrocytes via STAT1 and STAT5 intracellular pathways [83]. Leptin induced cell proliferation, proteoglycan and collagen synthesis in a biphasic fashion, with optimum concentration between 0.1 and 100 ng/mL—comparable to leptin levels in normal weight and obese individuals (average 40 ng/mL and 8 ng/mL, respectively) [81,83]. A similar study setting (culture of human knee cartilage) showed the expression of inducible nitric oxide synthase and production of nitric oxide (multipotent mediator, also involved in pain generation and transmission) at first after the co-stimulation with leptin and interferon-gamma via JAK2 intracellular pathway [84]. Another study with cultured human chondrocytes showed that leptin induced the expression of its receptor (LepR) [85]. Both leptin (concentration 0–1000 ng/mL) and transforming growth factor-β (TGF-β) induced cell proliferation and expression of aggrecan, GAGs, collagen type II and X. The stimulation was mediated by crosstalk of ERK, p38, and AKT signaling pathways, while TGF-β showed stronger proliferative and matrix-synthetic effect than leptin [85].

### 6.5. Animal Studies with IVD and Other Joints

In a study on mice, Gao et al. showed that at birth ≈ 89% of all NP cells were LepR positive. Their number slightly decreased, reaching a plateau at the age of one month (≈75% of cells were LepR positive) and remained stable until late adulthood [14]. Of note is that the pericellular matrix of LepR positive cells showed abundant immunostaining of aggrecan protein, while the staining of catabolic enzyme MMP-13 was found adjacent to LepR negative cells [14]. LepR positive cells started to “appear” in AF of mice after 14 postnatal days (primarily in the outer AF) and remained positive in the adulthood [14]. On the contrary, the notochordal cells of human NP were negative at LepR immunostaining [54].

There are also differences regarding leptin signaling in different animal models. For example, expression of LepRb in rat spinal growth plate showed gradual increase from early life, peaking at puberty, and declining afterwards [86]. On culture of rat NP, Miao et al. showed that leptin via LepRb—MAPK and JAK2/STAT3 pathway promoted expression of MMP-1 and MMP-13, and reduced expression of collagen type II, but not aggrecan [73]. Leptin also mediated through LepRb—PI3K/AKT pathway, but its effects were independent from the assessed catabolic enzymes. On the contrary, in cultured NP cells from scoliosis patients leptin decreased the expression of aggrecan and induced the expression of catabolic aggrecanases via p38-MAPK pathway [81].

In vitro study on isolated bovine NP and AF cells cultured with leptin showed an increase in levels of MMP-3 and MMP-9, and a trend for a decrease and production of GAGs [57]. Leptin caused the up-regulation of MMP-7, MMP-11, and TNF-α in AF cells, and aggrecanases, IL-6, and TNF-α in NP cells. Addition of IL-6 (an inflammatory mediator produced also in adipose tissue) into the medium markedly up-regulated TNF-α, IL-6, and aggrecanase 1. Importantly, leptin alone and in combination with TNF-a, IL-1ß, or IL-6 markedly increased the production of NO in NP cells. The authors concluded that “leptin can initiate degenerative processes and within the inflammatory environment seen in degenerate discs, it can potentiate degenerative process, thus supporting a biochemical link in the relationship between intervertebral disc degeneration, back pain, and obesity.” [57]. Similarly, leptin alone and by co-stimulation with IL-1ß enhanced the production of MMP-1, MMP-3, and MMP-13 in knee cartilage from patients with osteoarthritis [68].

An in vitro study on rat AF cells showed that leptin’s up-regulation of collagen type X and MMP-13 mRNA was increasing with increasing leptin concentration (from 1 ng/mL to 1 µg/mL) and also had stimulatory effect “on its own protein and receptor level” [87]. These leptin effects were mediated by p38 and ERK1/2 pathway. An inhibition of ERK1/2 implied that this pathway was important for stimulation of MMP-13 and collagen type X expression, but has minor role in the regulation of expression of leptin and LepR [87].

A rat model of LDD in vivo showed that increased leptin level in cartilage endplate coincide with its accelerated calcification [88]. In the same study in vitro experiments on cartilage endplate cells showed that leptin induced the expression of cartilage calcification marker genes (osteocalcin and runt-related transcription factor 2) in a dose-dependent manner, but not when the leptin concentration in medium was low (10 ng/mL). Further experiments with the highest leptin concentration (50 ng/mL) showed that leptin promotes osteogenic differentiation in cartilage endplate cells by intracellular signaling through ERK1/2 and STAT3. However, the STAT3 pathway seemed not to influence the assessed osteogenic genes in cartilage endplate. Furthermore, weekly administration of leptin to adult rats for six months (modeling the adiposity in adult age) caused neither microscopic ossification of cartilage endplate nor IVD degeneration. However, protein products of the two assessed osteogenic genes were increased [88]. The data on plasma leptin after chronic administration was not available. Altogether, Han et al. showed in rat model of LDD that high concentrations of leptin caused degeneration and ossification of cartilage endplate in in vitro and ‘degenerative alterations at the molecular level in vivo [88].

Another report showed that leptin stimulated rat chondrocyte proliferation and secretion of cartilage matrix components via p38-MAPK and AKT pathway [85]. In vivo experiment on rabbits with artificial knee cartilage defects showed that leptin-sustained release (via implanted hydrogel) induced cartilage regeneration [85].

A study of young mice with leptin receptor gene knockout (*Db/db*) showed that females develop severe alterations of the vertebral spine with the maintenance of large vacuolated notochordal cells, implying “delayed IVD cell proliferation and differentiation, rather than IVD degeneration” [89]. The authors suggested that impaired leptin signaling might have a protective role against diabetes- and obesity-induced IVD degeneration [89].

### 6.6. Leptin Signalung in Bone Marrow

The bone marrow is an important niche for leptin paracrine signaling. BM of adult mice harbors LepR positive multipotent mesenchymal stromal cells, which are the major sources of bone cells and adipocytes, as well as cartilage during bone healing in vivo [90]. Leptin binding with LepRb on human mesenchymal stem cells from bone marrow stimulated their proliferation and differentiation into osteoblasts [67]. Hence, the bone marrow niche houses a population of leptin-responsive multipotent cells able to differentiate into adipocytes, osteoblasts, and chondrocytes. After the enzymatic dissociation of mice vertebrae, it was shown that the LepR positive and collagen type II expressing osteoblasts increase until adulthood, representing about 60% of osteoblasts by the age of 10 months [90]. Thus, the presence of leptin in vertebral bone marrow niche is indispensable for the maintenance of vertebral bone. Of note, leptin deficiency has different effect on bone growth in the spine and femur, as well as differing effects on bone marrow on these locations [91].

## 7. Leptin and IVD Homeostasis

### 7.1. Leptin, Insulin, and IGF-1

The cells of IVD respond to anabolic and catabolic mediators diffusing through the IVD matrix. Human IVD cells in vitro showed DNA synthesis induction (as a proxy of cell proliferation) by medium conditioned by IVD cells [16]. Thus, the experiment confirmed autocrine/paracrine signaling route in IVD. Human IVD cells also respond to exogenous growth factors in vitro, e.g., via MAPK -ERK and PI-3K/AKT pathway after exposure to platelet-derived growth factor, basic fibroblast growth factor, and IGF-1 [16]. The same signaling pathway in IVD cells was induced with medium conditioned by IVD cells [16].

The cells of human AF are also responsive to these exogenous growth factors in vitro: IGF-1 and TGF-β induced the synthesis of collagen type II and proteoglycans [54]. The stimulation of IVD matrix synthesis was evident even in cultured AF cells from patients with advanced stage of LDD (Grade V in Thompson five-graded classification system of lumbar IVD degeneration based on gross morphology) [54].

In a culture of mice cartilage leptin induced the expression of IGF-1 receptor in chondrocytes and progenitor cells population, and herewith chondrocyte proliferation and differentiation [92]. Presence of IGF-1 (and TGF-β) was also directly demonstrated in NP of rat [93]. In bovine cartilage explants IGF-1 stimulated both aggrecan and collagen type II synthesis [94], as well as aggrecan synthesis in NP [82].

Numerous studies point at the importance and entanglement of crosstalk between insulin and leptin signaling pathways. For example, LepR gene knockout (*Db/db*) mice are among the most widely used models for studying diabetes mellitus type 2. *Db/db* mice developed IVD degeneration with high levels of MMP-3 in IVD matrix [82]. It is assumed that lack of leptin signaling and extensive diabetes-caused metabolic disturbances (inflammation, lipid and glucose metabolism) lead to IVD degeneration [82]. In the same study, IGF-1 treatment partially reversed the adverse effects of MMP-3 in LepR knockout mice [82]. A study on IGF-1 receptor gene knockout mice showed reduced collagen type II and aggrecan content and higher MMP-13 expression in IVD [95]. Another study on mice showed that leptin induces chondrocyte proliferation through increased expression of IGF-1 receptor [96]. A study on rabbit hippocampus showed that leptin increases the expression of IGF-1 via the JAK2/STAT5 pathway, while IGF-1 increases the expression of leptin [97]. A study on human AF cells from 25 patients (mean age 41.7 years, on average Thompson Grade III) with serum withdrawal (to mimic nutrient deficiency) showed antiapoptotic effect of IGF-1 and platelet-derived growth factor A [96].

Understanding the molecular interplay between insulin, leptin, IGF-1 and inflammation may be crucial in understanding the relation of IVD degeneration and obesity.

### 7.2. IVD and Leptin Paracrine Signaling

There are several tissue compartments that may take part in creating signaling milieu in a “long distance” paracrine fashion, i.e., by diffusion into the IVD. The nutrition of IVD relies on the diffusion through the cartilage endplate (Figure 1). It is unquestionable that leptin is produced in *vertebral* bone marrow, both in hematopoietic (“red”) bone marrow as well as in “yellow” MAT [19]. In the vertebral subchondral bone (bony endplate) of vertebrae are marrow contact channels and capillary bed (Figure 1). Subchondral bone also houses scarce adipocytes and immune cells—both able to secrete leptin [19].

Another potential source of leptin is epidural fat—the adipose tissue within the spinal canal. It was demonstrated that epidural fat expresses several cytokines [40,41].

The cells within IVD have the main role in short distance paracrine signaling. Leptin is present both within AF and within NP—where different subpopulations may act in synchrony. It was shown that within IVD exist LepR positive and LepR negative cells, both in mice [14], and in humans [75]. Abundant staining for main proteoglycan of IVD matrix was shown adjacent to LepR positive cells (in mice [14]). Thus, it is tempting to speculate that leptin may, under certain local circumstances, act as anabolic factor in IVD.

### 7.3. Experimental Pitfalls and Look Back

Most leptin studies are based on in vitro experiments on human and animal cells. It should be kept in mind that cell culture cannot mimic body environment entirely (e.g., regarding signaling molecules, organization of intercellular matrix, nutrient supply). Expression of genes and proteins might change during the pretreatment and passaging of cultured cells (e.g., enzyme treatment, washing, growing in bovine serum, use of antibiotics, etc.,). Moreover, the turnover of molecules involved in leptin signaling (including surface receptors) may be very fast [79]. Of note, the applied leptin concentrations in in vitro experiments did not correspond to the leptin levels in vivo in not just a few studies. For example, only high leptin levels in medium induced degeneration and ossification of rat cartilage endplate [88]. Even when serum leptin levels were established in mice in vivo (mean ≈ 1.0 ng/mL in male mice fed a high-fat chow), in in vitro experiments on cultured lymphocytes were used 20- and 100-fold higher leptin concentrations (and showed significant results) [64]. It was already observed that leptin’s anabolic effect on cultured chondrocytes (e.g., stimulation of cell proliferation and matrix synthesis) declines with increasing leptin levels (maximal effect with 10–100 ng/mL) [83]. It was speculated that higher levels of leptin might activate negative feedback mechanism [83].

Leptin is one of the hormones present in the IVD. Addition of just one or two mediators into the culture medium hardly corresponds the signaling milieu in vivo. Moreover, drawing conclusions on effects of leptin (or any other protein, e.g., MMPs) based on the presence of a protein in the culture, not taking account of its activity (e.g., presence of binding proteins, cleavage of signaling domain) may be far-fetched. The expression of catabolic enzymes (e.g., MMPs) upon in vitro stimulation (potentially using for IVD matrix unrealistically high concentrations), still does not confirm the leptins’ degenerative role, i.e., MMPs are often secreted with their inhibitors [17,98].

The implication that presence of LepR on cells of degenerated human IVD is directly related to negative effects of leptin—whose serum levels increase exponentially with an increasing volume of adipose tissue—likely oversimplifies the complex relationship between obesity, leptin pathway, and LDD. A great stumbling block is the uncertainty which isoform of LepR was demonstrated in a particular study, since the extracellular domains of the isoforms are reported to be identical [60,71]. Moreover, the extracellular domain is known to be heavily glycosylated, which can affect the downstream signaling [8,60,89]. Importantly, it was shown that majority of leptin receptors (80–90%) may reside in an intracellular compartment, without signaling capacity [60,71].

The regulation of leptin signaling is multilayered. The differential regulation of leptin-binding proteins (e.g., naturally present leptin-reactive immunoglobulins [67]) represents important level of control of leptin signaling. Leptin takes part in crosstalk with other mediators, with potentially additive, synergistic, or antagonistic effects. This crosstalk takes place at the level of cell membrane receptors, intracellular pathways, and regulation of transcription factors.

Tissue-specific regulation of LepR activity provides further level of control of leptin signaling. A great riddle is the presence of multiple isoforms of LepR—function of four out of five membrane-bound receptors is mostly unknown. The evolution does not maintain a structure without a reason. The differential regulation of leptin signaling pathways in different organs may shed light on few facets of molecular interplay between inflammation, insulin resistance, diabetes, and intervertebral disc degeneration. It is tempting to speculate that leptin may partly explain the molecular background of “the undefined”—metabolically healthy obese and metabolically unhealthy normal weight individuals.

### 7.4. Loves Me, Loves Me Not

Leptin—the most famous adipose tissue secreted hormone—in the human body is mostly observed in a negative connotation, as the hormone level increases with accumulation of body fat. Many studies, though mostly in in vitro and in animal models, confirmed leptin’s “bad reputation.” However, not few studies found that leptin may have protective role in IVD metabolism, e.g., it was implied that leptin stimulated IVD matrix synthesis [14] and NP cell proliferation [55,79]. Though, even regenerative and proliferative effects of leptin were interpreted in the light of leptin’s “bad reputation”—“leptin-induced cell proliferation might be a fundamental mechanism, underlying disc degeneration” [48].

Although leptin is considered as “the bad guy,” it is unquestionable that it has crucial role in bone and cartilage anabolism and regeneration.

## 8. Conclusions

IVD degeneration and IVD inflammation are two indiscerptible phenomena. Irrespective of underlying pathophysiological background (e.g., due to trauma, obesity, nutrient deficiency), the inflammation is crucial in triggering IVD degeneration. The proinflammatory cytokines seem to be of decisive importance. With advancement of IVD degradation, its cells increasingly release several proinflammatory mediators with nociceptive potential [3].

It is a common knowledge that leptin exerts pro-inflammatory effects in body. Numerous studies implied that leptin has detrimental effect on IVD homeostasis. However, several studies were convincing in demonstrating that leptin signaling in IVD has protective role and regenerative potential. These disagreements might be explained with unknown differences in architecture of signaling pathways. This review of the current literature suggests “back-to-basics approach”—we should first try to establish the nature of leptin signaling pathway in obesity, with focus on the intervertebral disc and its residents, before we try to repair something that maybe not *broken* at all.

## Figures and Tables

**Table 1:** Studies investigating leptin signaling in human intervertebral disc.

Authors and Year	Sample	Study Population	Key Findings
Gruber et al. 2007 [54]	AF in situ, NP	Disc tissue from 7 controls (aged newborn to 10 years) and 18 adults with LDD (surgical specimens)	Control group: uniform staining for leptin protein in AF and for leptin receptor. Lack of staining for leptin receptor of notochordal cells in NP. Patients with LDD: AF positive for LepR and leptin just for some cells.
Zhao et al. 2008 [55]	NP in situ	Disc tissue from 45 patients with lumbar disc herniation (aged 18–74 years).	Few leptin/LepR positive cells were single or paired, contrasting frequent positivity among clustered cells. Leptin and LepR positive cells in NP slightly increased with advancing age.
Hsu et al. 2020 [76]	NP in situ	Herniated disc tissue from 91 patient with LDD (mean age 58 years).	Positive staining for leptin in NP. Staining for matrix metalloproteinase-1 was the only independent parameter associated with the severity of IVD degeneration.
Koerner et al. 2014 [77]	AF extracts	IVD from 7 patients with LDD (mean age 43 years) and from 2 scoliosis patients. AF tissue separated into anterior and posterior AF.	The posterior AF leptin of LDD patients showed increased levels of 10 (of the 42 assessed) growth factors and cytokines (e.g., leptin, IL-4, IL-5, IL-6, TNF-β, and IGF 1).
Gao et al. 2020 [14]	NP filtered	Disc from 20 patients with LDD or scoliosis (mean age 36 years)	The percentage of LepR positive cells decreased with the increasing grade of IVD degeneration—from 75% in NP of patients with Grade II to 32% for Grade Ⅴ
Li et al. 2013 [78]	NP in vitro	Lumbar IVD from 45 patients with radiculopathy (mean age 48 years). Culture of NP from 8 patients with LDD (Thompson degeneration grade 2–3).	Both LepRa and LepRb mRNA were expressed in NP. LepRb mRNA expression correlated with body mass index. NP cells treated with leptin (10 ng/mL) showed increased expression of b-actin and vimentin as well as reorganization of F-actin.
Li et al. 2014a [75]	NP in vitro	IVD tissue from 7 patients with LDD (mean age 35 years, Pfirrmann grade 3)	Leptin (10 ng/mL) induced F-actin reorganization and stress fiber formation in cultured NP cells via the Rho/ROCK pathway.
Li et al. 2012 [79]	NP in vitro	IVD from 8 donors with LDD (31–40 years, Thompson grade 2–3).	NP cells express both LepRa and LepRb (mRNA). Time-dependent induction of NP cell proliferation by leptin in a concentration of 10 ng/mL (max. response at 96 h). Effects mediated through crosstalk between JAK/STAT3, PI3K/AKT, MAPK/ERK1/2 pathways.
Sun et al. 2018 [80]	*Ligamenta flava*	Ligamenta flava of 12 patients with lumbar spinal canal stenosis and 12 patients with lumbar disc herniation (mean age 58 years and 63 years).	Leptin (both the mRNA and protein) were higher in patients with lumbar spinal canal stenosis. Leptin induced the expression of collagen type I and type III, and IL-6 (via nuclear factor-κB pathway) (leptin concentration from 0 to 150 ng/mL).
Li et al. 2014b [81]	NP in vitro	IVD from 4 patients with idiopathic scoliosis (mean age 20 years, Thompson degeneration grade 1)	Treatment of NP cells with leptin (10 ng/mL) reduced aggrecan protein levels and increased the synthesis of aggrecanase-1 and -2 (maximal response at 48 h). Leptin at the concentration of 1000 ng/mL at the 48 h time maximally reduced aggrecan mRNA. The leptin effects were mediated via the p38-MAPK pathway.

AF—annulus fibrosus, IVD—intervertebral disc, NP—nucleus pulposus, LepR—leptin receptor, LDD—lumbar intervertebral disc degenerative disease, mRNA—messenger ribonucleic acid.

## Abbreviations

ADAMTS	a disintegrin and metalloproteinase with thrombospondin motifs
AF	annulus fibrosus
AKT	Akt kinase; protein kinase B
AMPK	adenosine monophosphate-activated protein kinase
BAT	brown adipose tissue
BMI	body mass index
CEP	cartilaginous endplate
CT	computed tomography
*Db/db*	leptin receptor gene knockout
ERK	extracellular signal-regulated kinase
GAGs	glycosaminoglycans
HDL	high-density lipoprotein
IGF-1	insulin-like growth factor 1
IL-	interleukin-
IVD	intervertebral disc
JAK2	Janus-activated kinase 2
LBP	low back pain
LDD	lumbar intervertebral disc degenerative disease
LDL	low-density lipoprotein
LepR	leptin receptor
JNK	c-Jun-N-terminal kinase
MAPK	mitogen-activated protein kinase
MAT	bone marrow adipose tissue
MHO	metabolically healthy but obese
MMP	matrix metalloproteinase
mRNA	messenger ribonucleic acid
MUNW	metabolically unhealthy normal weight
MRI	magnetic resonance imaging
NP	nucleus pulposus
PI3K	phosphatidylinositol 3-kinase
Rho/ROCK	Ras homolog gene family/Rho-associated coiled-coil-forming protein kinase
SAT	subcutaneous adipose tissue
STAT	signal transducer and activator of transcription
TGF-β	transforming growth factor-β
TNF-α	tumor necrosis factor-α
VAT	visceral adipose tissue
WAT	white adipose tissue
